# Effect of diffuse fraction on gross primary productivity and light use efficiency in a warm-temperate mixed plantation

**DOI:** 10.3389/fpls.2022.966125

**Published:** 2022-10-11

**Authors:** Peirong Liu, Xiaojuan Tong, Jinsong Zhang, Ping Meng, Jun Li, Jingru Zhang, Yu Zhou

**Affiliations:** ^1^ School of Ecology and Nature Conservation, Beijing Forestry University, Beijing, China; ^2^ Key Laboratory of Tree Breeding and Cultivation of State Forestry Administration, Research Institute of Forestry, Chinese Academy of Forestry, Beijing, China; ^3^ Key Laboratory of Water Cycle and Related Land Surface Processes, Institute of Geographic Sciences and Natural Resources Research, Chinese Academy of Sciences, Beijing, China

**Keywords:** gross primary productivity, light use efficiency, diffuse fraction, environmental factors, mixed plantation

## Abstract

Diffuse radiation (*I*_f_) is one of important variables determining photosynthetic rate and carbon uptake of forest ecosystems. However, the responses of gross primary productivity (GPP) and light use efficiency (LUE) to diffuse fraction (DF) are still poorly understood. We used a 6-year dataset of carbon flux at a warm-temperate mixed plantation site in North China to explore the impacts of DF on GPP and LUE. During 2011-2017, ecosystem apparent quantum yield (α) and photosynthesis at photosynthetically active radiation (PAR) of 1800 µmol m^-2^ s^-1^ (*P*_1800_) on cloudy days were 63% and 17% higher than on clear days, respectively. Under lower vapor pressure deficit (VPD) and air temperature (*T*_a_) conditions, canopy photosynthesis was significantly higher on cloudy skies than on clear skies. On half-hourly scale, increased DF enhanced α and *P*_1800_. Daily GPP peaked at a median DF (=0.5), while daily LUE significantly increased with DF (*p*<0.01). Both GPP and LUE were mainly controlled directly by DF and PAR. DF had an indirect effect on LUE and GPP mainly through PAR. At high DF levels (>0.5), the increase in LUE did not make GPP enhancement. The direct effect of DF on GPP and LUE under lower *T*_a_ and VPD was more sensitive than under higher *T*_a_ and VPD. When DF was incorporated into the Michaelis-Menten model, it performed well in the GPP estimation, and the determination coefficient increased by 32.61% and the root mean square error decreased by 25.74%. These findings highlight the importance of incorporating DF into carbon sequestration estimation in North China.

## Introduction

GPP is an important indicator to characterize CO_2_ uptake by ecosystems photosynthesis (Chapin et al., 2011) and accounts for the largest CO_2_ flux in the carbon cycle of terrestrial ecosystems (Beer et al., 2010). Forest ecosystems contribute around 40–50% of terrestrial GPP flux, which is one of important components of terrestrial carbon cycle (Cai et al., 2014). Solar radiation affects plant growth and terrestrial ecosystem productivity directly (Knohl and Baldocchi, 2008; Mercado et al., 2009). It is reported that solar radiation has decreased by 15–30% in certain areas of the Northern Hemisphere since 1970 (Stanhill and Cohen, 2001; Zhang et al., 2011). Ecophysiological processes, especially carbon cycle, will be affected by the change of solar radiation in the future (Wang et al., 2008). Cloudiness and aerosols in the atmosphere reduce solar radiation but increase the diffuse fraction (DF) (Roderick et al., 2001; Oliphant et al., 2011; Yang et al., 2013).

Previous observational experiments have widely concluded that DF has an important influence on GPP and light use efficiency (LUE) in terrestrial ecosystems (Alton et al., 2007; Kanniah et al., 2013; Durand et al., 2021). The difference between direct and diffuse radiation impacts leaf photosynthesis generally depends on specific species and environmental conditions (Berry and Goldsmith, 2020), but direct radiation usually promotes more leaf photosynthesis under high radiation (Durand et al., 2021). Increased DF can enhance canopy photosynthesis, increasing LUE at ecosystem scales (Zhang et al., 2011; Wang et al., 2015; Wang et al., 2018). The mechanism of DF photosynthesis enhancement is mainly that, on one hand, the cloudiness leads to a more uniform distribution of light in the canopy, enhancing the photosynthesis of sunlit and shaded leaves (Steiner and Chameides, 2005; Park et al., 2018). On the other hand, *I*_f_ from all directions can easily reach the bottom of the canopy, thereby improving canopy photosynthesis considerably (Urban et al., 2012; Kanniah et al., 2013). The increase in DF was accompanied by changes to light quality and quantity, which resulted in higher DF suppressing GPP in three forest ecosystems (Zhang et al., 2011), a temperate poplar plantation (Xu et al., 2017) and boreal coniferous and mixed forests (Ezhova et al., 2018). Moreover, the influence of DF on GPP and LUE in terrestrial ecosystems is related to vegetation type, canopy structure and leaf area index (LAI) (Alton et al., 2007; Knohl and Baldocchi, 2008; Kanniah et al., 2012; Park et al., 2018). The increasing *I*_f_ substantially improves CO_2_ uptake of the forests with large LAI (Roderick et al., 2001; Zhang et al., 2011), whereas the effect of *I*_f_ on carbon uptake in short or sparse vegetation is not significant (Niyogi et al., 2004; Letts et al., 2005; Park et al., 2018). Mean leaf tilt angle, canopy height and LAI dominated canopy structure and enhanced canopy heterogeneity, which improved canopy photosynthesis (Emmel et al., 2020). Therefore, it is necessary to clarify how the two opposing effects of GPP respond to DF in different ecosystems.

Both GPP and LUE were correlated with environmental factors at the ecosystem scale, such as air temperature (*T*_a_) (Yamori et al., 2014), vapor pressure deficit (VPD) (Yuan et al., 2019) and soil water content (SWC) (Liu et al., 2020a). DF indirectly influences environmental factors to affect canopy photosynthesis (Kanniah et al., 2012; Han et al., 2019). By increasing DF, solar radiation is distributed evenly throughout the canopy, reducing *T*_a_ and VPD, and thus improving canopy photosynthesis (Zhang et al., 2011; Xu et al., 2017). Other studies, however, indicated that *T*_a_ and VPD had little effect on regulating the ecosystem photosynthesis response to *I*_f_ (Jing et al., 2010; Kanniah et al., 2011; Oliphant et al., 2011). Changes in environmental conditions may affect GPP response to *I*_f_ of forest and grassland ecosystems (Xu et al., 2017; Li et al., 2020). The coupling between DF and environmental factors will have complex effect on ecosystem photosynthesis (Kanniah et al., 2013; Gui et al., 2021). To date, our knowledge on the direct and indirect effect of DF on LUE and GPP is still limited, especially considering the constraints of environmental conditions.

LUE models have been developed to estimate photosynthetic production and investigate the impacts of environmental stresses on photosynthetic production (Nichol et al., 2010; Wang et al., 2017). Most LUE models treat vegetation canopy as a big single-leaf, and productivity linearly increases with the amount of incoming photosynthetically active radiation (PAR) (Pei et al., 2022). To estimate the effect of solar radiation on GPP accurately, considering the division of solar radiation into direct and diffuse parts in GPP simulations, such as MM_dif_ Model (Cai et al., 2009), DIFFUSE Model (Donohue et al., 2014) and DTEC GPP Model (Yan et al., 2017). A top-down model of canopy photosynthesis (MM_dif_ model) was developed by Cai et al. (2009) after the effect of DF on GPP was added into the Michaelis-Menten (MM) model, and there were lower systematic errors in GPP estimation on clear and cloudy days using the MM_dif_ model. The effect of DF on GPP varies greatly in different ecosystems, influencing the accuracy of LUE models (Yuan et al., 2014; Wang et al., 2015; Yang et al., 2019). By effectively assessing the effect of DF on the accuracy of simulated GPP, and provides a scientific basis for subsequent improvement of LUE models. Here, we employed a big single-leaf model apprehending total and diffuse radiation control GPP physical mechanism under different environmental conditions.

Plantations cover about 80 million ha, which is 36% of the total forests in China (China's Forestry Administration, 2018). The magnitude of carbon sequestration by planted forests was 47.8% of carbon sink of total forests in China during 1977–2008 (Guo et al., 2013). In north China, planted forests of the hilly region play a vital ecological barrier and carbon sink (Fang et al., 2007; Tong et al., 2012). Up to date, carbon sequestration and water use efficiency of plantations have been explored in this region (Tong et al., 2012; Guo et al., 2013; Tong et al., 2014; Xue et al., 2016; Xu et al., 2018). However, the impact of DF on LUE and GPP is not well understood. We hypothesize that the indirect effects of DF on temperate forest ecosystem LUE and GPP under different environmental conditions are mainly caused by PAR. Based on a 6-year carbon flux dataset of a mixed plantation, we used the path analysis method and a big single-leaf model to reveal the potential mechanisms of DF on LUE and GPP. To evaluate the performance of the MM_dif_ model combining DF into the GPP estimation.

## Materials and methods

### Site description

CO_2_ flux and micrometeorological variables were measured at Xiaolangdi Forest Ecosystem Research Station of Jiyuan, Henan Province, China (36°01′N, 112°28′E, 410 m a.s.l.). The site is located at the south of the Taihang Mountain and the north of the Yellow River, with a warm-temperate continental monsoon climate. Annual average temperature is 13.4°C and the annual precipitation is 642 mm in recent three decades. During the growing season (April-September), the prevailing wind direction is the northeast. The dominant tree species is cork oak (*Quercus variabilis*), with an age of 47, average canopy height of 11.6 ± 1.2 m and average diameter at breast height of 16.8 ± 3.3 cm. Other two species are black locust (*Robinia pseudoacacia L.*) and arborvitae (*Platycladus orientalis*), with 43 and 45 years old, and average canopy heights of 10.5 ± 2.1 and 9.2 ± 1.6 m, respectively. The soil is classified as brown loam with high gravel content. Much detailed information of this site is reported by Tong et al. (2012).

### Measurements of carbon flux and meteorological variables

Carbon flux was measured by the eddy covariance system with a 3-D sonic anemometer (Model CSAT3, Campbell Scientific Inc., USA) and an open-path and fast response infrared CO_2_/H_2_O analyzer (Model Li-7500, Li-COR Inc., USA) at the height of 30 m above the surface. Raw data were collected at a frequency of 10 Hz, and the flux data were recorded by a data logger (Model CR5000, Campbell Scientific Inc., USA).

Air temperature and humidity were monitored by psychrometers (Model HMP45C, Campbell Scientific Inc., USA). A Global solar radiometer (Model CM11, Kipp and Zonen Inc., NL) was installed at the 27 m height. PAR was measured by a quantum sensor (Model LI190SB, Li-COR Inc., USA). Direct solar radiation has been monitored by a radiometer (Model CSD3, Kipp and Zonen Inc., NL) since 2016. Soil moisture at the depths of 0, 5, 10 and 20 cm was monitored by time domain reflectometry (TDR) probes (Model CS615-L, Campbell Scientific Inc., USA). Soil temperature sensors were placed at the depths of 5, 10 and 20 cm. Additionally, precipitation was measured. All above data were sampled by the data loggers (Model CR10XT and CR23XTD, Campbell Scientific Inc., USA) at 5-min intervals. Observed data from the 2011 to 2017 growing seasons has been used, except for 2015.

### Flux data proceeding

Half hourly CO_2_ flux data was corrected by WPL algorithm (Webb et al., 1980) and 2-D coordination rotation (McMillen, 1988). CO_2_ flux would be underestimated at night due to weak turbulence, and it should be deleted when u_*_ was lower than the threshold (0.35 m s^-1^) (Tong et al., 2012). The data exceeded three times of the average variance value were removed. Due to instrument malfunction and unfavorable meteorological conditions, flux data should be deleted. During 2011-2017, the mean availability of valid CO_2_ flux data was 66.7%, 34.3%, and 50.7% for daytime, nighttime and total, respectively. The small data gap (<2 h) was filled with the linear interpolation method (Falge et al., 2001). For the large gaps (>2 h), missing daytime fluxes were interpolated by using mean diurnal variation (MDV) with a 14-day moving window, and nighttime data gaps were filled by an exponential equation (Falge et al., 2001).

### Calculation of LUE

GPP was calculated as:


(1) $$GPP=NEP+R_{\text{ec}}$$

where NEP is net ecosystem productivity and it is measured by the eddy covariance system. *R*_ec_ is ecosystem respiration, and it was estimated as:


(2) $$R_{\text{ec}}=R_{0}\times Q_{10}^{\left(T_{\text{s}}/10\right)}$$

where *R*_0_ is the base ecosystem respiration rate when soil temperature is at 0°C, *T*_s_ is soil temperature at the depth of 10 cm, *Q*_10_ is temperature sensitivity coefficient for *R*_ec_ and it represents respiration rate rising with every 10°C increment of temperature. The nighttime *R*_ec_ (i.e. the nighttime net ecosystem carbon exchange) values were used to estimate *R*_0_ and *Q*_10_.

At the ecosystem level, LUE is the ratio of GPP to PAR reaching above the canopy:


(3) $$LUE=\frac{GPP}{PAR}$$

### Definition of clear skies

Clearness index (CI) can be used to represent the effect of the atmosphere on extraterrestrial radiation (*I*_0_), and it is defined as:


(4) $$CI=\frac{I_{g}}{I_{0}}$$

where *I*_g_ is global radiation (W m^-2^), *I*_0_ is the extraterrestrial radiation at a plane parallel to the earth surface (W m^-2^) (Gu et al., 1999):


(5) $$I_{0}=I_{sc}\left[1+0.033\text{cos}\left(\frac{360t_{d}}{365}\right)\right]\text{sin}\beta$$

where *I*_sc_ is solar constant (1370 W m^-2^), *t*_d_ is the day of the year, *β* is solar elevation angle:


(6) sinβ=sinϕsinδ+cosϕcosδcosω

where *ϕ* is local latitude, *δ* is solar declination and *ω* is hour angle.

DF was estimated using the BRL-1 model (Liu et al., 2020b):


(7) $$DF=\frac{1}{1+\text{exp}\left(a_{0}+a_{1}CI+a_{2}AST+a_{3}\text{sin}\beta +a_{4}CI_{d}+a_{5}\psi +a_{6}RH\right)}$$

where *a*_0_, *a*_1_, *a*_2_, *a*_3_, *a*_4_, *a*_5_ and *a*_6_ are fitted parameters, *AST* is apparent solar time, RH is relative humidity, CI_d_ is daily CI and *ψ* is a persistence of global radiation:


(8) $$C{\text{I}}_{\text{d}}=\frac{\sum_{i=1}^{n}{\text{I}}_{\text{g}}}{\sum_{i=1}^{n}I_{0}}$$

(9) $$\psi =\{\begin{array}{ll} \frac{CI_{\text{t}+1}+CI_{\text{t}-1}}{2} & \text{sunrise}<\text{t}<\text{sunset}\\ CI_{\text{t}+1} & \text{ t}=\text{sunsrise}\\ CI_{\text{t}-1} & \text{ t}=\text{sunset} \end{array}$$

where t is time, *n* is daylight hours.

**Figure 1:**
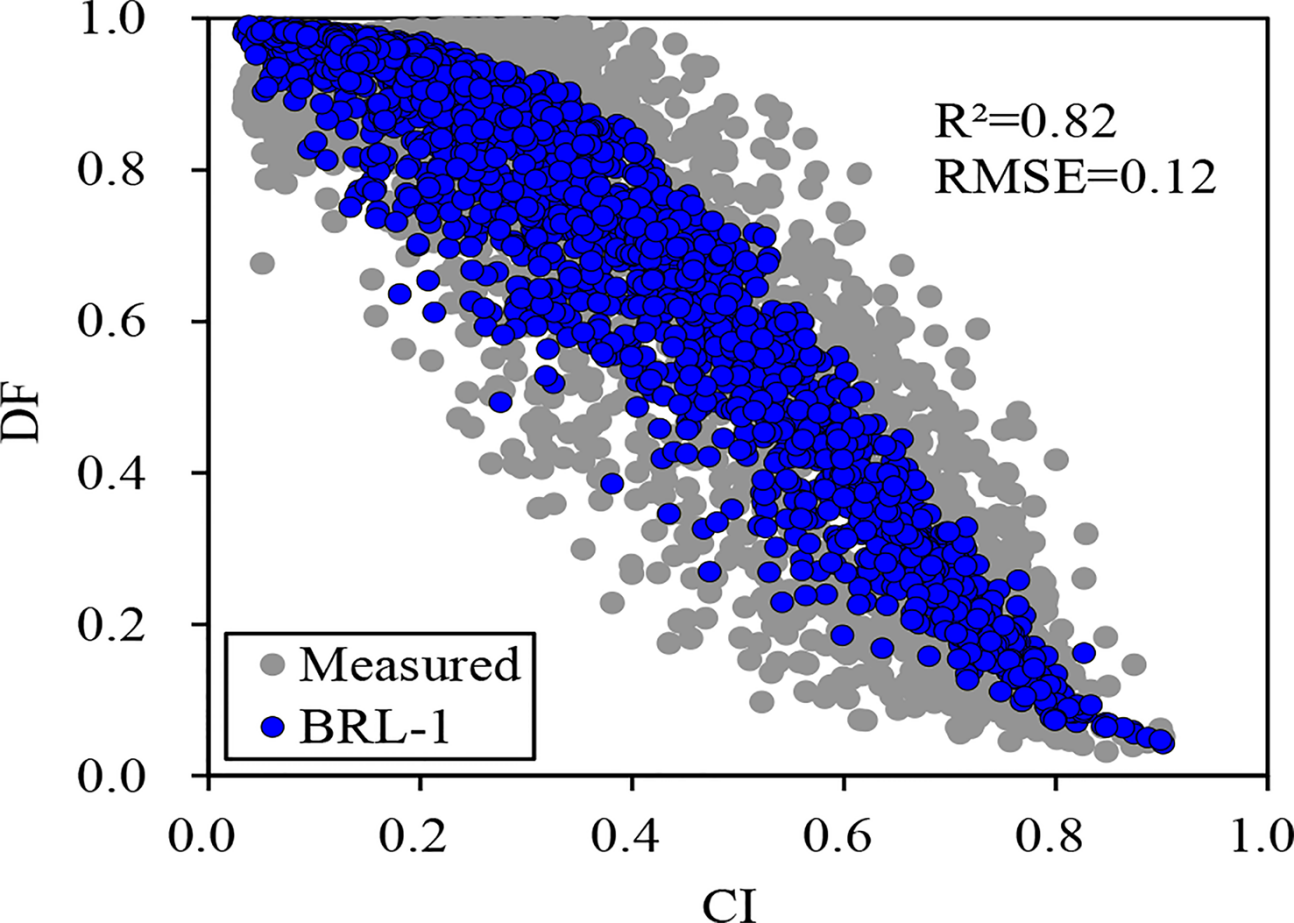
Scatter plot between clearness index (CI) and measured, simulated (BRL-1 Model) diffuse fraction (DF).

The scatter plots of both measured and simulated DF with CI in 2017 are shown in **Figure 1**. The BRL-1 model performed well at 95% confidence level. During the period from 2011 to 2014, DF was estimated using the BRL-1 model. Diffuse radiation (*I*_f_) and direct radiation (*I*_r_) were calculated:


(10) $$I_{\text{f}}=I_{\text{g}}\times DF$$

(11) $$I_{\text{r}}=I_{\text{g}}-I_{\text{f}}$$

TD_f_ is the ratio of diffuse PAR to PAR (Spitters, 1986; Gu et al., 1999):


(12) $$TD_{\text{f}}=\frac{\left[1+0.3\left(1-q^{2}\right)\right]q}{1+\left(1-q^{2}\right)\cos^{2}\left(90^{{}^{\circ}}-\beta \right)\cos^{3}\beta }$$

(13) $$q=I_{\text{f}}/I_{\text{g}}/CI$$

We calculated diffuse PAR (PAR_f_) and direct PAR (PAR_r_) as:


(14) $$PAR_{\text{f}}=PAR\times TD_{\text{f}}$$

(15) $$PAR_{\text{r}}=PAR-PAR_{\text{f}}$$

Average CI (CI_a_) was estimated at the half-day time scale, and the 2-month running mean CI_2m_ was calculated. Clear skies were defined: CI_a_≥1.2CI_2m_; CI increased with sin*β* smoothly. We used the coefficient of 1.2 to make the proportion of clear skies to cloudy skies close to the long-term results of meteorological observations.

### Ecosystem photosynthesis-light response models

The response of ecosystem photosynthesis to PAR was fitted by the rectangle hyperbola equation (Michaelis and Menten, 1913):


(16) $$\begin{array}{ll} GPP=\frac{\alpha P_{\max}PAR}{\alpha PAR+P_{\max}} & \text{MM model} \end{array}$$

where α is the ecosystem apparent quantum yield, *P*_max_ is the maximum ecosystem photosynthetic capacity (mg CO_2_ m^-2^ s^-1^). GPP under low light intensity (*P*_600_ at PAR=600 µmol m^-2^ s^-1^) was compared with that under strong light intensity (*P*_1800_ at PAR=1800 µmol m^-2^ s^-1^). DF was incorporated into the MM model (Cai et al., 2009):


(17) $$\begin{array}{ll} GPP=\frac{\alpha P_{\text{max}}\left(PAR_{f}+kPAR_{r}\right)}{\alpha (PAR_{f}+kPAR_{r})+P_{\text{max}}} & {\text{MM}}_{\text{dif}}\text{model} \end{array}$$

where (PAR_f_ +*k*PAR_r_) is the effective incident PAR, *k* is a measure of the contribution of PAR_r_ to effective incident PAR, and it ranges from 0 to 1. The MM_dif_ model can be expressed as the MM model when *k* equals to 1. The α, *P*_max_ and *k* values were fitted by the nonlinear Gauss-Newton algorithm. The differential responses of canopy photosynthesis to PAR_f_ and PAR_r_ were included in the MM_dif_ model.

GPP had quadratic functions with *T*_a_ and VPD, and it peaked at the optimum of *T*_a_=30 °C and VPD=1.5 kPa (data not shown). Therefore, we analyzed the relationship between GPP and PAR under different sky conditions according to the classes VPD ≤ 1.5 kPa and VPD>1.5 kPa, and *T*_a_ *≤* 30 °C and *T*_a_>30°C classes. SWC was divided into water-stressed conditions (SWC ≤ 15%) and non-water-stressed conditions (SWC>15%).

Root mean squared error (RMSE), the relative error (RE) and the determination coefficient (R^2^) were used to compare model performances:


(18) $$\text{RMSE}=\sqrt{\frac{\sum_{\text{i}=1}^{\text{n}}{\left(E_{\text{i}}-M_{\text{i}}\right)}^{2}}{n}}$$

(19) $$\text{RE}=\frac{M_{\text{i}}-E_{\text{i}}}{M_{i}}\times 100\%$$

(20) $$R^{2}=\frac{\left(\sum_{\text{i}=1}^{\text{n}}\left(E_{\text{i}}-\bar{E_{\text{i}}}\right)\left(M_{\text{i}}-\bar{M_{\text{i}}}\right)\right)}{\sum_{\text{i}=1}^{\text{n}}{\left(E_{\text{i}}-\bar{E_{\text{i}}}\right)}^{2}\sum_{\text{i}=1}^{\text{n}}{\left(M_{\text{i}}-\bar{M_{\text{i}}}\right)}^{2}}$$

where n is the data number, *E*_i_ and *M*_i_ are the simulated and measured values, respectively.

### Path analysis

Path analysis was used to investigate the direct and indirect effect of environmental variables on GPP and LUE. It is a multiple regression model, which can deal with the casual relationships among correlated variables (Shipley, 2004):


(21) $$\begin{array}{ll} r_{\text{i,y}}=r_{\text{i},1}P_{1,\text{y}}+r_{\text{i},2}P_{2,\text{y}}+\cdots +r_{\text{i,i}}P_{\text{i,y}}+\cdots +r_{\text{i,n}}P_{\text{n,y}} & \left(\text{i}=1,2,3,\cdot \cdot \cdot ,\text{n}\right)\;\;\; \end{array}$$

where i is different independent variables, *r*_i,y_ is the correlation coefficient between the independent variable i and the dependent variable y, *r*_i,n_ is the correlation coefficient between different independent variables, *P*_i,y_ is the direct effect of the independent variable i on the dependent variable y (standardized regression coefficient), and *r*_i,n_×*P*_n,y_ (i≠n) is the indirect effect of independent variable i affecting another independent variable n which in turn affects the dependent variable y.

We used DF, PAR, *T*_a_, VPD and SWC in path analysis. Path analysis was performed by SPSS AMOS software (version 24.0, IBM Inc., USA). All input variables initially need to be standardized. Maximum likelihood method is applied in the calculation. Output results included direct impact (SDE, [-1,1]), indirect impact (SIE, [-1,1]) and total impact (STE, [-2,2]). Positive and negative values indicated positive and negative effects, respectively, and the absolute value of coefficient represented relative effect among variables.

## Results

### Micrometeorological variables

**Figure 2:**
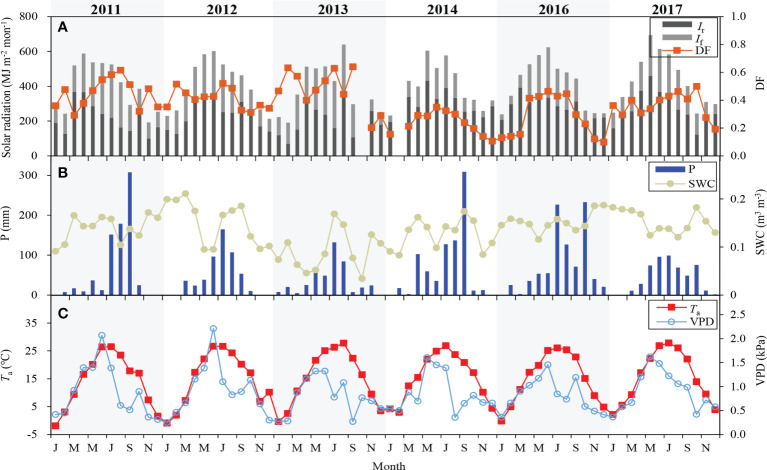
Monthly variations of **(A)** diffuse radiation (*I*_f_), direct radiation (*I*_r_), diffuse fraction (DF), **(B)** precipitation (P), soil water content (SWC), **(C)** air temperature (*T*_a_) and vapor pressure difference (VPD).

*I*_g_ peaked in May-June in most years, except that it was highest in August in 2013 (**Figure 2A**). Annual *I*_g_ in the years of 2011-2014 was lower than that in 2016 and 2017. *I*_f_ peaked in June-July, and it varied from 939 to 1606 MJ m^-2^ in the growing season, between 206 and 566 MJ m^-2^ in the non-growing season. Monthly mean DF ranged from 0.10 to 0.64 during the 6-year period. Annual mean DF was about 0.41, and it was the largest in 2013 (0.52) and the lowest in 2014 (0.28). The strongest *I*_g_ in August of 2013 led to its highest mean *T*_a_ in the same period. However, in the other five years, monthly mean *T*_a_ peaked in June-July (**Figure 2C**). Annual mean *T*_a_ was lowest in 2011 (14.0°C) and highest in 2017 (15.6°C).

Compared to the other five years, annual precipitation was highest (888 mm) in 2016 and lowest (437 mm) in 2013 (**Figure 2B**). During the 6-year period, annual precipitation was 654 ± 168 mm, close to the value (642 mm) in the recent 30-year. The average SWC in the growing season was largest (0.15 m^3^ m^-3^) in 2012 and lowest (0.09 m^3^ m^-3^) in 2013 owing to low precipitation. Monthly mean VPD peaked in May of 2014 and 2017, June of 2011, 2012, 2013 and 2016 due to low precipitation and high temperature in the same period (**Figure 2C**). Strong solar radiation and less precipitation were responsible for a higher mean VPD (1.24 kPa) in 2012.

### Seasonal patterns in GPP and LUE

**Figure 3:**
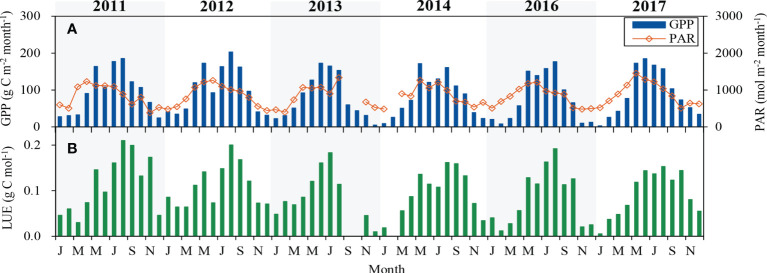
Seasonal variation in monthly **(A)** gross primary productivity (GPP) and photosynthetically active radiation (PAR), **(B)** light use efficiency (LUE) during the 6-year period.

Monthly average GPP, PAR and LUE during the 6-year period are shown in **Figure 3**. In spring, GPP increased with the increase of PAR and *T*_a_ (**Figures 1**, **3**). With the decline of PAR and *T*_a_ in autumn, GPP also dropped gradually. The maximum of monthly GPP appeared in late spring of 2014, but it occurred in summer of the other five years, ranging from 172 to 203 g C m^-2^ month^-1^. The amount of GPP ranged between 925 g C m^-2^ in 2016 and 1209 g C m^-2^ in 2012. In 2016, low PAR and DF limited photosynthesis and therefore resulted in a reduction in GPP. Annual GPP of 2012 was higher than the other five years, which may be due to stronger PAR and larger SWC.

Monthly LUE varied from 0.01 to 0.21 g C mol^-1^, and the maximum monthly average LUE occurred in July-August during the 6-year period. Annual mean LUE was highest (0.11 g C mol^-1^) in 2011 and 2012 and lowest (0.09 g C mol^-1^) in 2013, 2016 and 2017. During the 6-year period, the mean annual LUE was 0.10 ± 0.01 g C mol^-1^.

### Diurnal patterns in GPP and LUE

**Figure 4:**
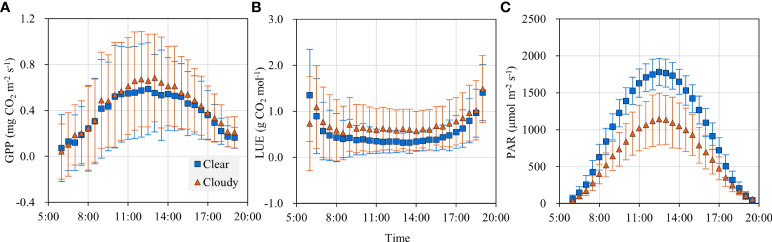
Diurnal variations of **(A)** gross primary productivity (GPP), **(B)** light use efficiency (LUE) and **(C)** photosynthetically active radiation (PAR) under cloudy and clear sky conditions.

The diurnal patterns of GPP under clear and cloudy conditions are shown in **Figure 4A**. GPP was about 0.12 mg CO_2_ m^-2^ s^-1^ in the early morning and late afternoon, and it increased with solar radiation and peaked at noon. GPP was 13.93% higher under cloudy skies than under clear skies during the period of 10:00 am-14:00 pm. Though the overall amount of PAR reaching the canopy was normally lower under cloudy sky conditions (**Figure 4C**), there was an increase in GPP when the main component of the radiation moved from direct to diffuse above the canopy.

LUE was high in the early morning and late afternoon (**Figure 4B**). It was that PAR decreased more rapidly than GPP during the transition from light to dark (**Figure 4C**). During the period of 8:00 am-17:00 pm, LUE was low and maintained a value of 0.29 g CO_2_ mol^-1^ in the clear skies and 0.48 g CO_2_ mol^-1^ in the cloudy skies. LUE was 66% larger in the cloudy skies than in the clear skies.

### Light response of GPP under different sky and environment conditions

**Figure 5:**
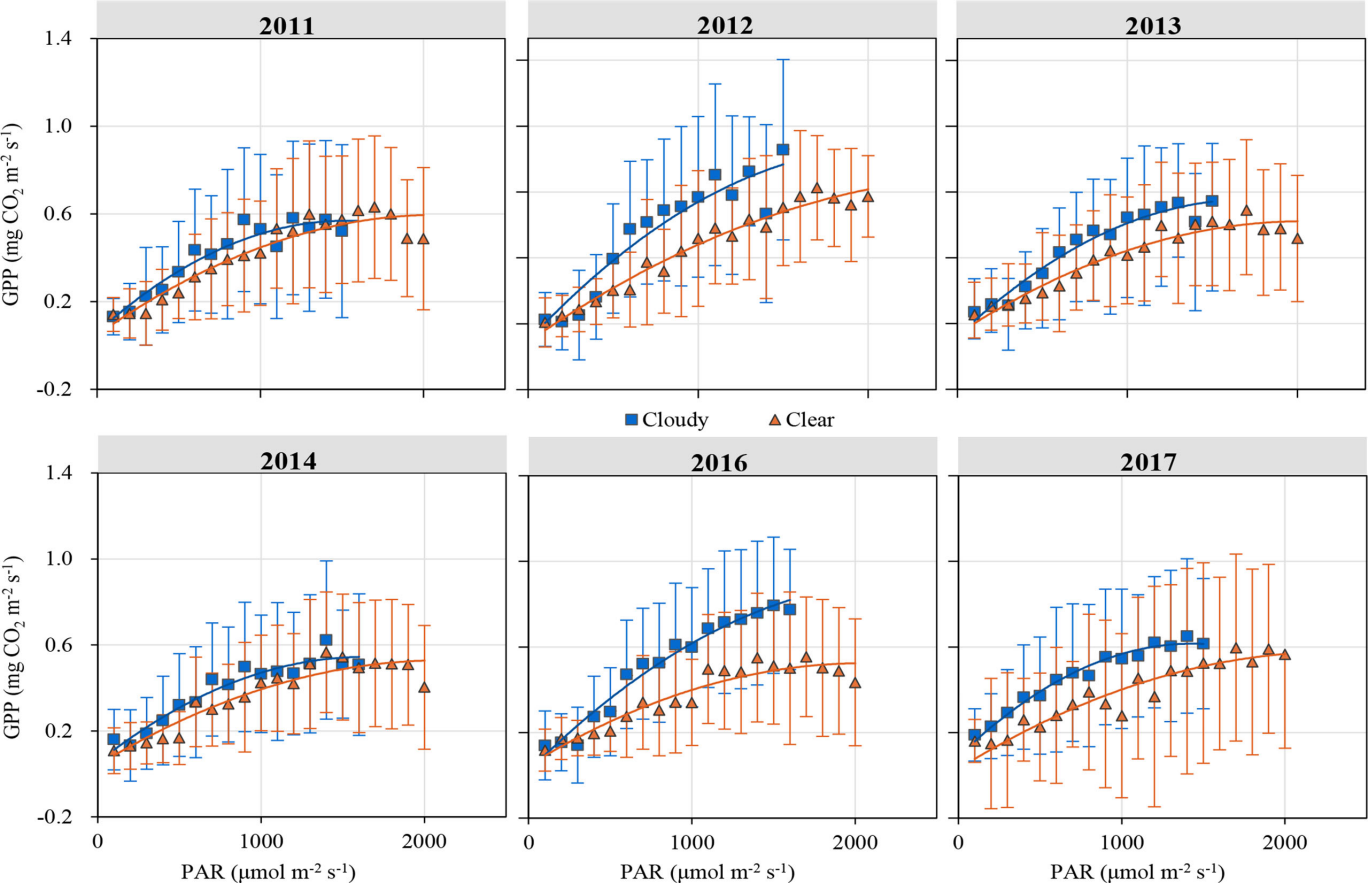
Light response curves of half-hourly gross primary productivity (GPP) to photosynthetically active radiation (PAR) under different sky conditions. All data with standard error bars are averaged at a 100 µmol m^-2^ s^-1^ interval under the same sky condition.

**Figure 5** illustrates the response of GPP to PAR under clear and cloudy sky conditions during the growing season. Half-hourly GPP was averaged by PAR at a 100 µmol m^-2^ s^-1^ interval. At the same PAR level, GPP was larger in cloudy skies than in clear skies. Light response parameters (α and *P*_max_), *P*_600_ and *P*_1800_ derived from Eq.(16) are shown in **Table 1**. Compared with clear sky conditions, the values of α, *P*_600_ and *P*_1800_ under cloudy sky conditions increased by 16–132%, 18–57% and 5–71%, with an average enhancement of 54%, 36% and 27%, respectively. The *P*_max_ values ranged from 0.84 to 1.95 mg CO_2_ m^-2^ s^-1^ under cloudy sky conditions, and from 0.82 to 1.64 mg CO_2_ m^-2^ s^-1^ under clear sky conditions. During the 6-year period, *P*_max_ under cloudy skies was 21% higher than under clear skies. α, *P*_600_ and *P*_1800_ values under higher DF (0.8≤DF<1.0) were 198–202%, 82–115% and 19–54% higher than under lower DF (DF<0.2 and 0.2≤DF<0.4). α, *P*_600_ and *P*_1800_ values significantly increased with DF (*p*<0.05).

**Table 1:** Light response parameters estimated by the MM model under different sky conditions.

Year	Sky condition	α	*P*_max_(mg CO_2_ m^-2^ s^-1^)	*P*_600_(mg CO_2_ m^-2^ s^-1^)	*P*_1800_(mg CO_2_ m^-2^ s^-1^)	R^2^	n
2011	Clear	0.019	0.96	7.47	13.30	0.61	1273
	Cloudy	0.027	0.87	8.83	14.00	0.50	1019
2012	Clear	0.015	1.64	7.07	15.38	0.72	824
	Cloudy	0.023	1.89	10.28	20.86	0.70	486
2013	Clear	0.019	0.89	7.31	12.71	0.59	1093
	Cloudy	0.024	1.16	9.27	16.30	0.57	728
2014	Clear	0.016	0.86	6.56	11.77	0.60	1359
	Cloudy	0.023	0.84	8.10	13.16	0.48	1020
2016	Clear	0.018	0.82	6.73	11.75	0.55	1402
	Cloudy	0.020	1.95	9.57	20.05	0.67	1162
2017	Clear	0.015	1.03	6.47	12.50	0.56	1286
	Cloudy	0.035	0.87	10.14	15.05	0.52	707
2011–2017	Clear	0.016	1.04	6.73	12.84	0.70	7237
	Cloudy	0.026	0.97	9.16	15.00	0.55	5122
DF interval	<0.2	0.009	1.48	4.59	10.81	0.44	1020
	0.2–0.4	0.014	1.26	6.61	13.57	0.55	2611
	0.4–0.6	0.019	1.10	7.78	14.41	0.64	2117
	0.6–0.8	0.021	1.23	8.58	15.97	0.69	2258
	>0.8	0.027	1.12	9.85	16.63	0.63	4353

All regressions are significant at the level of *p*<0.05. DF is diffuse fraction, *α* is ecosystem apparent quantum yield, *P*_max_ is the maximum ecosystem photosynthetic capacity, *P*_1800_ is GPP at high PAR (1800 µmol m^−2^ s^−1^) and *P*_600_ is GPP at low PAR (600 µmol m^−2^ s^−1^).

**Figure 6:**
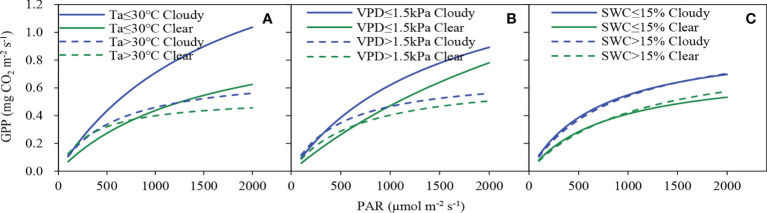
Light response curves between half-hourly gross primary productivity (GPP) and photosynthetically active radiation (PAR) in clear and cloudy skies under different **(A)** air temperature (*T*_a_), **(B)** vapor pressure deficit (VPD) and **(C)** soil water content (SWC) conditions.

The responses of GPP to PAR under different *T*_a_, VPD and SWC classes were calculated to consider the co-varying nature of environmental factors (**Figure 6**). Compared with clear sky conditions, α values under cloudy sky conditions increased by 54%, 71% and 42% at lower *T*_a_, VPD and SWC, 38% and 47% at higher VPD and SWC, respectively. Under cloudy sky conditions, the *P*_1800_ value increased by 65%, 17% and 31% at lower *T*_a_, VPD and SWC, and by 22%, 12% and 23% at higher *T*_a_, VPD and SWC, respectively. At the mixed planted forest stand, the sensitivity of canopy photosynthesis to the change of SWC was low when the sky was covered by clouds. Lower *T*_a_ and VPD under cloudy sky conditions resulted in larger α and *P*_1800_ values (**Figure 6** and **Table 2**).

**Table 2:** Light response parameters estimated by the MM model under different environmental conditions.

Environmental condition	Sky condition	α	*P*_max_(mg CO_2_ m^-2^ s^-1^)	*P*_600_(mg CO_2_ m^-2^ s^-1^)	*P*_1800_(mg CO_2_ m^-2^ s^-1^)	R^2^	n
*T*_a_ ≤ 30°C	Clear	0.016	1.10	0.31	0.60	0.65	5342
	Cloudy	0.025	1.93	0.50	0.99	0.66	4329
*T*_a_>30°C	Clear	0.036	0.53	0.34	0.45	0.39	1895
	Cloudy	0.029	0.72	0.37	0.55	0.40	793
VPD ≤ 1.5 kPa	Clear	0.014	2.27	0.31	0.73	0.71	2951
	Cloudy	0.023	1.59	0.44	0.85	0.65	3312
VPD>1.5 kPa	Clear	0.023	0.68	0.32	0.49	0.51	4286
	Cloudy	0.031	0.70	0.38	0.55	0.45	1810
SWC ≤ 15%	Clear	0.021	0.75	0.32	0.52	0.59	3738
	Cloudy	0.029	0.96	0.43	0.68	0.54	2426
SWC>15%	Clear	0.018	0.90	0.31	0.55	0.59	3499
	Cloudy	0.027	1.00	0.41	0.68	0.55	2696

All regressions are significant at the level of *p*<0.05. *T*_a_ is air temperature, VPD is vapor pressure deficit and SWC is soil water content. α is ecosystem apparent quantum yield, *P*_max_ is the maximum ecosystem photosynthetic capacity, *P*_600_ is GPP at low PAR (600 µmol m^−2^ s^−1^) and *P*_1800_ is GPP at high PAR (1800 µmol m^−2^ s^−1^).

### Effects of DF on GPP and LUE

**Figure 7:**
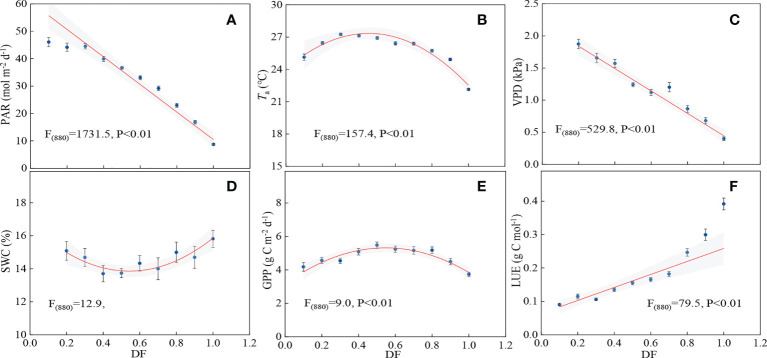
Relationship between **(A)** photosynthetically active radiation (PAR), **(B)** air temperature (*T*_a_), **(C)** vapor pressure deficit (VPD), **(D)** soil water content (SWC), **(E)** gross primary productivity (GPP), **(F)** light use efficiency (LUE) and diffuse fraction (DF).

The changes of LUE and GPP with DF are shown in **Figures 7E, F**. LUE increased significantly with increasing DF (*p*<0.01), and the values of LUE increased from 0.09 to 0.39 g C mol^-1^, implying that cloud conditions were beneficial for LUE. GPP had a remarkable quadratic relationship with DF, and it peaked when DF was about 0.5 (*p*<0.01). It was indicated that partly cloudy sky conditions were favorable for carbon uptake. Both PAR and VPD decreased linearly with increasing DF (**Figures 7A, C**). At high DF (>0.5), *T*_a_ and SWC had significant decreasing and increasing trends, respectively (**Figures 7B, D**). Water conditions improved at high DF (>0.5), but the decreasing PAR and *T*_a_ mainly limited canopy photosynthesis, and hence the maximal GPP occurred with an optimal DF (0.5). Carbon assimilation increased when PAR was lower than 33.03 mol m^-2^ d^-1^, and any reduction in PAR may reduce GPP when light intensity is less than this value (**Figures 7A, E**).

### Direct and indirect influences of environmental factors on GPP and LUE

The path analysis of the total and direct effect of environmental variables on GPP and LUE is illustrated in **Table 3**. There were significant correlations between GPP and PAR, DF, *T*_a_ and VPD during the growing season. PAR and DF had significant predictive power to predict the GPP value. LUE was significantly correlated with PAR, DF and VPD (*p*<0.05), but it was primarily regulated by DF. The negative direct impact of VPD on GPP and LUE was evident under higher *T*_a_ and VPD conditions, and the small positive direct impact of *T*_a_ was found for lower *T*_a_ and VPD conditions. Compared with higher *T*_a_ and VPD conditions, the direct effect of PAR on GPP was more pronounced under lower *T*_a_ and VPD conditions. Under water or temperature-limited conditions, both GPP and LUE were mainly controlled by PAR and VPD. In addition, SWC had little effect on LUE and GPP. It is likely because the deep root system of the forest can supply sufficient water for top soil when water in the top soil is depleted (Wu et al., 2012; Wang et al., 2018), and SWC did not limit plant photosynthesis at this site. The direct effect of DF on GPP and LUE was greater in low *T*_a_ and VPD than in high *T*_a_ and VPD.

**Table 3:** Total effect (TE) and direct effect (DE) of environmental factors on gross primary productivity (GPP) and light use efficiency (LUE).

	Environmental condition	PAR		DF		VPD		*T*_a_		SWC	
		TE	DE	TE	DE	TE	DE	TE	DE	TE	DE
GPP	*T*_a_ *≤* 30°C	0.59	0.78	-0.26	0.23	0.17	-0.12	0.22	0.09	0.08	0.06
	*T*_a_>30°C	0.50	0.50	-0.21	–	-0.31	-0.37	-0.03	0.14	0.29	0.21
	VPD ≤ 1.5 kPa	0.62	0.78	-0.24	0.27	0.28	0.01	0.27	0.07	0.09	0.05
	VPD>1.5 kPa	0.54	0.60	-0.23	0.08	-0.18	-0.27	-0.03	0.08	0.19	0.16
	SWC ≤ 15%	0.63	0.80	-0.24	0.26	0.24	-0.04	0.22	0.04	0.09	–
	SWC>15%	0.62	0.76	-0.25	0.28	0.32	0.03	0.30	0.09	0.09	0.06
LUE	*T*_a_ *≤* 30°C	-0.30	-0.07	0.39	0.31	-0.25	-0.07	-0.08	0.04	0.09	0.09
	*T*_a_>30°C	-0.37	-0.32	0.34	0.08	-0.35	-0.33	-0.07	0.12	0.29	0.25
	VPD ≤ 1.5 kPa	-0.24	-0.05	0.34	0.32	-0.16	–	–	0.05	0.05	0.08
	VPD>1.5 kPa	-0.34	-0.24	0.35	0.17	-0.25	-0.23	-0.06	0.08	0.19	0.20
	SWC ≤ 15%	-0.31	-0.07	0.41	0.29	-0.32	-0.14	-0.22	–	–	0.04
	SWC>15%	-0.34	-0.15	0.39	0.25	-0.25	-0.15	-0.09	0.13	0.16	0.13

PAR is photosynthetically active radiation, *T*_a_ is air temperature, VPD is vapor pressure deficit and SWC is soil water content. “–” means that it is insignificant. Others are significant at the level of *p*<0.05.

**Table 4** shows the indirect effect of DF on GPP and LUE, which describes how GPP and LUE are affected by DF via other environmental factors. DF primarily interacted with PAR to affect GPP and LUE. Under high *T*_a_ conditions, DF positively interacted with VPD and PAR to impact GPP and LUE. The indirect effect of DF through *T*_a_ and SWC on GPP and LUE was not significant. Compared with higher *T*_a_ and VPD conditions, the indirect effect of DF through PAR on GPP and LUE was more significant under lower *T*_a_ and VPD conditions.

**Table 4:** The indirect effect from diffuse fraction (DF) through other environmental factors to gross primary productivity (GPP) and light use efficiency (LUE).

Environmental condition	Indirect effect from DF via	PAR	VPD	*T*_a_	SWC
*T*_a_ *≤* 30°C	GPP	-0.534	0.062	-0.017	0.000
	LUE	0.045	0.034	-0.007	-0.002
*T*_a_>30°C	GPP	-0.289	0.122	-0.033	-0.002
	LUE	0.181	0.111	-0.029	-0.006
VPD ≤ 1.5 kPa	GPP	-0.502	-0.001	-0.007	-0.004
	LUE	0.033	0.003	-0.005	-0.007
VPD>1.5 kPa	GPP	-0.363	0.062	-0.004	-0.009
	LUE	0.140	0.056	-0.004	-0.013
SWC ≤ 15%	GPP	-0.479	0.056	-0.004	-0.012
	LUE	0.112	0.007	0.061	0.007
SWC>15%	GPP	-0.564	0.093	-0.059	0.005
	LUE	0.119	0.039	-0.040	0.008

PAR is photosynthetically active radiation, *T*_a_ is air temperature, VPD is vapor pressure deficit and SWC is soil water content. The values are significant at the level of *p*<0.05.

### Comparison of GPP estimated by the MM and MM_dif_ models

**Figure 8:**
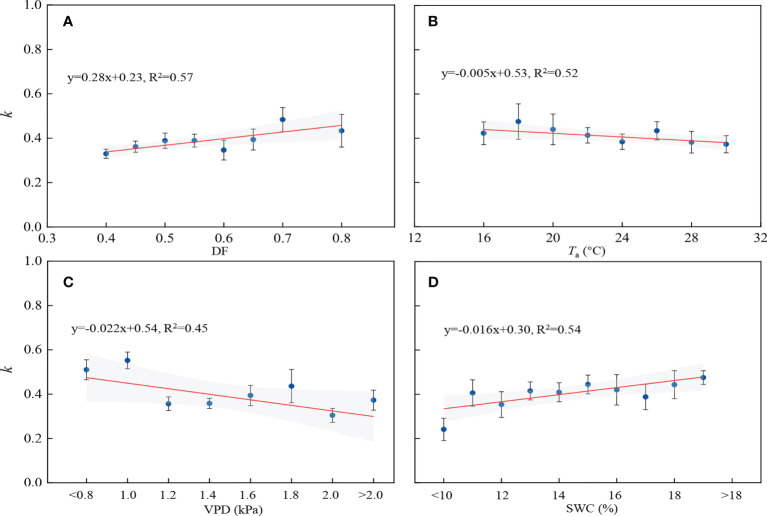
Relation between the parameter *k* of the MM_dif_ model and **(A)** diffuse fraction (DF), **(B)** air temperature (*T*_a_), **(C)** vapor pressure deficit (VPD) and **(D)** soil water content (SWC).

The MM_dif_ and MM ecosystem photosynthesis-light response models were used to estimate GPP during the growing season. The parameter *k* of the MM_dif_ model ranged between 0.12 and 0.62, with an average of 0.38. The low *k* indicated the significant impact of *I*_f_ on GPP and the fraction of light-limited sunlit leaves. The relationships between *k* and DF, *T*_a_, VPD and SWC are shown in **Figure 8**. The *k* value significantly increased with DF and SWC. Conversely, higher *T*_a_ and VPD reduced the *k* value. Forest canopies receipted more PAR_r_ under cloudy sky conditions with higher SWC, lower *T*_a_ and VPD, which was beneficial to canopy photosynthesis.

**Figure 9:**
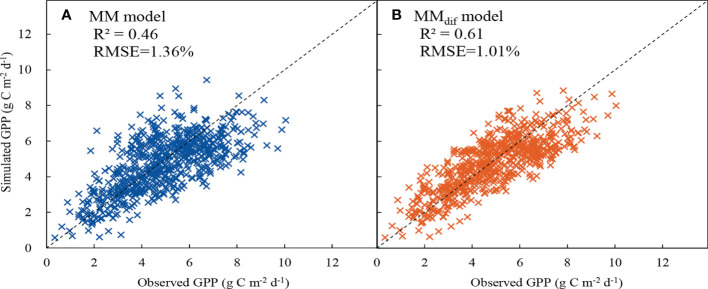
Comparison between measured gross primary productivity (GPP) vs. simulated values from **(A)** MM and **(B)** MM_dif_ models. The dashed line is 1:1 line.

**Figure 10:**
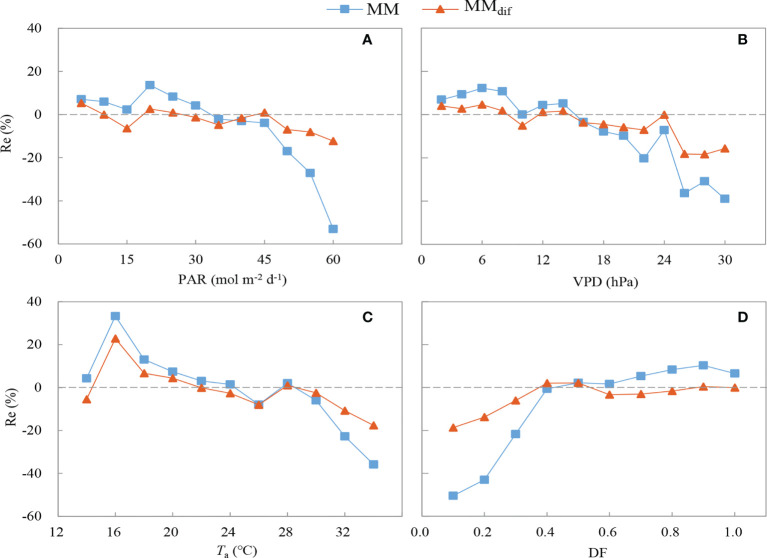
Relative error of the MM and MM_dif_ models under different environmental conditions: **(A)** photosynthetically active radiation (PAR), **(B)** vapor pressure deficit (VPD), **(C)** air temperature (*T*_a_) and **(D)** diffuse fraction (DF).

The MM_dif_ model performed better than the MM model (**Figure 9**). The R^2^ value increased by 32.61% and the RMSE decreased by 25.74% after the impact of DF on GPP was included in the MM_dif_ model. The RE of the MM and MM_dif_ models were compared under different environmental conditions (**Figure 10**). In general, the estimates of GPP produced by the MM_dif_ model were less biased than the MM model under different environmental conditions. Compared with the MM_dif_ model, the MM model significantly overestimated GPP value at high PAR, *T*_a_, VPD and low DF levels. Under high and low DF conditions, the performance of the MM_dif_ model was better than that of the MM model. This discrepancy may be because the MM model tends to represent median DF conditions. The RE of MM_dif_ and MM models were 1.5–1.8% and 5.6–7.0% in low *T*_a_ and VPD, respectively. In case for high *T*_a_ and VPD, the RE of MM_dif_ and MM models were respectively -14.2–9.2% and -29.2–19.3%. These results employed that ecosystem photosynthesis-light response model performed better after DF was incorporated into the GPP simulation.

## Discussion

### Response of photosynthesis to different sky conditions

Canopy photosynthesis increased under cloudy sky conditions (Gu et al., 2002; Knohl and Baldocchi, 2008; Zhang et al., 2011; He et al., 2013; Ezhova et al., 2018; Zhou et al., 2021). Under clear sky conditions, sunlit leaves of the canopy receive more direct solar radiation, causing photosynthesis saturation. However, shaded leaves receive low solar radiation under clear sky conditions and are sensitive to radiation changes (Roderick et al., 2001; Gu et al., 2002; Alton et al., 2007). The enhancement of LUE was 36% under cloudy skies (**Figure 4B**), close to the results reported for the tropical broadleaf (33%) (Alton et al., 2007), higher than obtained in a sparse canopy (6–18%) (Alton et al., 2007), but lower than the crops (110%) (Choudbury, 2001) and the old-growth temperate forest (50%) (Hollinger et al., 1994). The value of α under cloudy sky conditions was 63% higher than under clear sky conditions (**Table 1**), close to the finding of Rocha et al. (2004) in a northern hardwood forest. Under thick cloud conditions, α increased by 21% in a tropical savanna forest (Kanniah et al., 2013).

In the mixed plantation, GPP significantly enlarged on cloudy days in comparison to clear days (**Figures 5**, **6**). The values of *P*_600_ and *P*_1800_ were 36% and 17% larger under cloudy skies than those under clear skies, respectively (**Table 1**). On cloudy days, the α and *P*_n750_ values of a subtropical coniferous plantation increased by 19.2% and 23.4%, respectively (Han et al., 2019). Under different environmental classes, the values of α and *P*_1800_ were also higher on cloudy days than on clear days (**Table 2**). Under cloudy sky conditions, the vertical distribution of PAR in the whole forest canopy was more even and additional *I*_f_ reaches the below canopy, and hence enlarging photosynthetic rates of shaded leaves (Kanniah et al., 2011; Urban et al., 2012; Dengel et al., 2015). Moreover, the blue/red light ratio is high under cloudy sky conditions, which is conducive to stimulating photochemistry and stomatal opening (Matsuda et al., 2004; Dengel and Grace, 2010; Urban et al., 2012). On cloudy days, the enhancement of α and *P*_1800_ in low *T*_a_ and VPD were higher than in high *T*_a_ and VPD (**Table 2**). It is similar to those results that were found in a poplar plantation (Xu et al., 2017) and a desert steppe (Li et al., 2020). Under cloudy sky conditions, low *T*_a_ and VPD were more conducive to enhancing canopy photosynthesis. It was because water or temperature-unlimited conditions reduce stomatal resistance and promote leaf carbon dioxide uptake (Zhang et al., 2021).

### Impacts of DF on LUE and GPP

Previous studies reported that DF enhanced ecosystem photosynthesis in a broad-leaved forest (Oliphant et al., 2011), a subtropical coniferous forest (Han et al., 2019), and three forest canopies (a sparse boreal needle-leaved, a temperate broad-leaved, and a dense tropical broad-leaved) (Alton et al., 2007). Increasing DF led to a large enhancement of photosynthesis at light levels (**Table 2**). LUE was positively correlated with DF, and DF explained 34–41% of the variation in LUE (**Table 3**). It was found that LUE had positive linear relations with DF in a poplar plantation (Xu et al., 2017) and a wheat cropland (Yang et al., 2019). Increased DF improved photosynthetic rate by improving photosynthesis of shade leaves but limiting light saturation of sunlit leaves (Gu et al., 2002). Higher DF corresponded to a large value of *k* because increasing clouds reduced the limitation of strong light on ecosystem photosynthesis (**Figure 8**). GPP had a significant relationship with DF, and 8–28% of the variation in GPP could be explained by DF (**Table 3**), consistent with the finding in a temperate deciduous beech forest (Wang et al., 2018). Similarly, DF explained 41% and 17% of seasonal GPP variation in a cropland and temperate forest, respectively (Cheng et al., 2015).

Though the interaction between DF and PAR enhanced LUE, GPP decreased due to low PAR. GPP initially increased and then decreased with increasing DF, and it peaked under moderate cloudy sky conditions (DF=0.5) in the mixed plantation (**Figure 7**). In forests, croplands and global FLUXNET sites, GPP peaked at a median of DF (Oliphant et al., 2011; Huang et al., 2014; Yang et al., 2019; Zhou et al., 2021). It is indicated that an increase of clouds may weaken the “diffuse fertilization effect” when light intensity is lower than a specified level. The path analysis showed that DF regulated GPP mainly by the indirect effect of radiation (**Table 4**). The quantity of radiation is still a critical factor for GPP despite a higher fraction of *I*_f_ can enhance LUE (Alton et al., 2007; Kanniah et al., 2013). Due to the reduction in PAR (**Figure 7**), LUE enhancement under diffuse sunlight was insufficient to increase GPP at a high DF level (>0.5). Therefore, the net effect of DF on GPP depends on the balance between the increase in photosynthesis for shade leaves resulting from the rise of the diffuse fraction of the PAR and the weakening of the photosynthetic rates for sunlit leaves due to reducing total PAR (Mercado et al., 2009; Kanniah et al., 2013). Moreover, compared with cloud water droplets, the scattering of aerosols prefers for forward direction, and it is more conservative with respect to *I*_g_ (Farquhar and Roderick, 2003; Alton et al., 2007). In recent years, the increasing aerosols due to human activities and climate change have enlarged the photosynthetic rate (Zhang et al., 2021). Changes of clouds and aerosols will become the source of uncertainty affecting carbon sink of forest ecosystems in the future (Boucher et al., 2013; Melnikova and Sasai, 2020). In addition, the responses of LUE and GPP to increasing DF depend on plant species, canopy structure, LAI and environmental factors (Kanniah et al., 2012; Cheng et al., 2015). The forest with stratified layers canopy was much more tightly correlated with *I*_f_ (Cheng et al., 2015) than croplands and grasslands with open canopies (Hollinger et al., 1994; Niyogi et al., 2004). Plant LAI varies seasonally, and leaf nitrogen increases with LAI, which further influences the changes in canopy photosynthesis (Reich, 2012). The effect of DF on photosynthesis increased with seasonally increasing LAI in the deciduous broadleaf forest (Knohl and Baldocchi, 2008) and the evergreen coniferous forest (Cheng et al., 2015).

Different environmental conditions may affect the response of GPP to cloudiness in forest ecosystems (Park et al., 2018; Gui et al., 2021). The direct effects of environmental factors showed that the increase of GPP and LUE responses to DF was influenced by the discrepancy in the effect of different *T*_a_ and VPD on canopy photosynthesis (**Table 3**), and the role of cloudiness in regulating GPP and LUE needs to be taken into different environmental conditions. Under water (VPD ≤ 1.5 kPa) or temperature (*T*_a_ *≤* 30 °C)-unlimited conditions, the effect of cloudiness on canopy photosynthesis was more obvious in the mixed plantation (**Figure 6**). The average value of *k* decreased under water or temperature-limited conditions (**Figure 8**), indicating that extreme environmental factors reduced the effective incident radiation demand for photosynthesis. Under high VPD conditions, stomatal closure avoids water loss but limits canopy photosynthetic rate (Körner, 1995). *T*_a_ can promote ecosystem photosynthesis because increased *T*_a_ enhances enzyme activity and photosynthetic electron transfer efficiency (Berry and Bjorkman, 1980), but high *T*_a_ above an optimum can also limit photosynthetic rates (June et al., 2004). These results revealed that the promotion of canopy photosynthesis by DF reached its maximum when water or temperature conditions were unlimited. High temperatures and wet summers as well as heat stress may limit the response of GPP and LUE to cloudiness (**Figure 2** and **Table 3**). Furthermore, under VPD>1.5 kPa or *T*_a_>30°C conditions, high VPD was the important factor restricting GPP in the mixed plantation (**Table 3**). It is demonstrated that the primary environmental element limiting GPP changed with the environmental conditions.

### Comparison of GPP derived from the MM and MM_dif_ models

Quantitative estimation of GPP is necessary for understanding the response of terrestrial ecosystems to the change in the quality of incoming radiation (Wang et al., 2015; Yan et al., 2020). The MM_dif_ model described the response of canopy photosynthesis to light as a nonlinear process. In the GPP estimation, the MM_dif_ model performed better than the MM model when DF was incorporated into the MM_dif_ model (**Figure 9**). The MM_dif_ model can be regarded as a single big-leaf model, it avoids some errors of the previous single big-leaf models of canopy photosynthesis (Sellers et al., 1996). A previous study found that the performance of a joint “top-down” GPP and ET model was improved when the cloudiness index was taken into the simulation, and RMSE decreased by 11.7% in GPP for a high latitude temperate deciduous forest (Wang et al., 2018). The LUE model significantly underestimated GPP on cloudy days due to ignoring the effect of DF on canopy photosynthesis (Yuan et al., 2014). Similarly, if the impact of *I*_f_ is ignored, the LUE model will underestimate GPP increasing trend in China (Yan et al., 2020). Half-hourly CO_2_ flux is measured over a large flux footprint, making the MM_dif_ model effective for overcoming the spatial heterogeneity of canopy radiation regime and suitable for simulating canopy photosynthesis at the regional scale (Cai et al., 2009). Additionally, SIF (solar-induced chlorophyll fluorescence) is also usually used to estimate GPP and it is an optical signal directly emitted by plants during the light reactions of photosynthesis (Wu et al., 2022). Cloudy sky conditions affect SIF-GPP relationships by enhancing photosynthesis in light-limited leaves (Yang et al., 2018). The canopy escape fraction increases with PAR_f_ fraction due to the variation of near-infrared reflectance and radiation (Kim et al., 2021). Therefore, the connection between different sky conditions and the SIF-GPP relationships should not be neglected in improving the estimation of terrestrial GPP.

There are differences in the simulation performance of the MM and MM_dif_ models under different environmental conditions in this study. Under different DF conditions, GPP estimated by the MM_dif_ model was much closer to the measured GPP in comparison with that determined by the MM model. It was attributed to the inclusion of direct and diffuse components in the MM_dif_ model (**Figure 10D**), consistent with the results in a hemisphere bog (Goodrich et al., 2015) and a deciduous forest (Wang et al., 2018). There are lower systematic errors in GPP estimated by the MM_dif_ model under clear and overcast sky conditions (Cai et al., 2009). Compared with the MOD17 model, the CI-LUE model less underestimated GPP under cloudy sky conditions after cloudiness was incorporated into the simulation (Wang et al., 2015). Since PAR_r_ and PAR_f_ were simulated separately, the MM_dif_ model performs well under lower *T*_a_ and VPD conditions (**Figures 10B, C**). At higher *T*_a_ and VPD, the weak effect of DF and PAR on GPP may lead to limit the performance of the MM_dif_ model. We found that MM_dif_ model significantly overestimated GPP value in high *T*_a_ and VPD, which may affect the applicability of LUE models in areas with extreme environmental conditions. LUE models should quantify environmental stresses to realistically capture environmental controls on ecosystem functions. Because the effect of environmental variables on photosynthesis varies in different ecosystems (Gui et al., 2021), more studies under various environment conditions are needed to identify the optimal parameters of the LUE models. Moreover, water factors have complex effects on GPP simulation, so multiple indicators may be needed to capture the diverse responses of plants to water stress (Pei et al., 2022). Besides environmental factors, leaf optical parameters (reflectance and transmittance) influence the response of GPP to DF (Durand et al., 2021). Therefore, GPP will be estimated accurately if these associated variables are incorporated into the simulation in the future.

## Conclusions

We explored the impact of DF on GPP and LUE of a temperate mixed plantation in North China during a 6-year period. Both GPP and LUE were larger under cloudy sky conditions than under clear sky conditions at the half-hourly scale. On cloudy days, the enhancement of α and *P*_1800_ in low *T*_a_ and VPD was higher than in high *T*_a_ and VPD. DF explained 34–41% and 8–28% of the variation in LUE and GPP, respectively. GPP initially increased and then decreased with increasing DF, and it peaked under moderate cloudy sky conditions (DF=0.5). Daily LUE significantly increased with DF. Meanwhile, PAR was the major intermediate variable of the regulation of DF on LUE and GPP. The trade-off effect between DF and PAR on GPP is linked with sky conditions, canopy structure and environmental conditions. In low *T*_a_ and VPD, canopy photosynthesis is more easily increased with DF in the mixed plantation. These findings highlight the importance of incorporating DF into GPP estimation, and the performance of the MM_dif_ model in estimating GPP was better than that of the MM model, especially under low *T*_a_ and VPD conditions. Under future climate change and human activities, the responses of ecosystem productivity to sky and environmental conditions are nonlinear. Thus, additional factors (e.g. aerosols, vegetation phenology, leaf optical parameters and environmental factors) interacting with GPP should be taken into account in the GPP simulation.

## Data availability statement

The original contributions presented in the study are included in the article/supplementary material. Further inquiries can be directed to the corresponding authors.

## Author contributions

PL and XT carried out the data processing and analysis and wrote the manuscript. JSZ and PM contributed to the conception and design of the study. PL and JL organized the data and performed the statistical analysis. JRZ and YZ carried out data collection. All authors participated the manuscript editing and approved the final version. All authors contributed to the article and approved the submitted version.

## Funding

This study was sponsored by the National Natural Science Foundation of China (31872703; 31570617) and the National Key R & D Program of China (2020YFA0608101).

## Acknowledgments

We thank Dr. WenwenYuan and Mr. Yongbin Huang for their assistance with field measurements and instrumentation maintenance.

## Conflict of interest

The authors declare that the research was conducted in the absence of any commercial or financial relationships that could be construed as a potential conflict of interest.

## Publisher’s note

All claims expressed in this article are solely those of the authors and do not necessarily represent those of their affiliated organizations, or those of the publisher, the editors and the reviewers. Any product that may be evaluated in this article, or claim that may be made by its manufacturer, is not guaranteed or endorsed by the publisher.

## References

[B1] Alton P. B. North P. R. Los S. O. (2007). The impact of diffuse sunlight on canopy light-use efficiency, gross photosynthetic product and net ecosystem exchange in three forest biomes. Global Change Biol. 13, 776–787. doi: 10.1111/j.1365-2486.2007.01316.x

[B2] Beer C. Reichstein M. Tomelleri E. Ciais P. Jung M. Carvalhais N.. (2010). Terrestrial gross carbon dioxide uptake: Global distribution and covariation with climate. Science 329, 834–838. doi: 10.1126/science.1184984 20603496

[B3] Berry J. Bjorkman O. (1980). Photosynthetic response and adaptation to temperature in higher plants. Annu. Rev. Plant Physiol. 31, 491–543. doi: 10.1146/annurev.pp.31.060180.002423

[B4] Berry Z. C. Goldsmith G. R. (2020). Diffuse light and wetting differentially affect tropical tree leaf photosynthesis. New Phytol. 225, 143–153. doi: 10.1111/nph.16121 31418864

[B5] Boucher O. Randall D. Artaxo P. Bretherton C. Feingold G. Forster P.. (2013). “Clouds and aerosols//Climate change 2013: The physical science basis,” in Contribution of working group I to the fifth assessment report of the intergovernmental panel on climate change (New York: Cambridge University Press).

[B6] Cai T. Black A. Jassal R. S. Morgenstern K. Nesic Z. (2009). Incorporating diffuse photosynthetically active radiation in a single-leaf model of canopy photosynthesis for a 56-year-old Douglas-fir forest. Int. J. Biometeorol. 53, 135–148. doi: 10.1007/s00484-008-0196-x 19132410

[B7] Cai W. Yuan W. Liang S. Liu S. Dong W. Chen Y.. (2014). Large Differences in terrestrial vegetation production derived from satellite- based light use efficiency models. Remote Sens. 6, 8945–8965. doi: 10.3390/rs6098945

[B8] Chapin F. S. Matson P. A. Vitousek P. (2011). Principles of terrestrial ecosystem ecology (Berlin: Springer Science & Business Media).

[B9] Cheng S. J. Bohrer G. Steiner A. L. Hollinger D. Suyker A. Phillips R. P.. (2015). Variations in the influence of diffuse light on gross primary productivity in temperate ecosystems. Agr. Forest Meteorol. 201, 98–110. doi: 10.1016/j.agrformet.2014.11.002

[B10] China's Forestry Administration (2018) Forest resources of China 2014–2018. Available at: http://www.forestry.gov.cn.

[B11] Choudbury B. J. (2001). Estimating gross photosynthesis using satellite and ancillary data: Approach and preliminary results. Remote Sens. Environ. 75, 1–21. doi: 10.1016/S0034-4257(00)00151-6

[B12] Dengel S. Grace J. (2010). Carbon dioxide exchange and canopy conductance of two coniferous forests under various sky conditions. Oecologia 164, 797–808. doi: 10.1007/s00442-010-1687-0 20577763

[B13] Dengel S. Grace J. MacArthur A. (2015). Transmissivity of solar radiation within a *Picea sitchensis* stand under various sky conditions. Biogeosciences 12, 3825–3853. doi: 10.5194/bg-12-4195-2015

[B14] Donohue R. J. Hume I. H. Roderick M. L. McVicara T. R. Beringeref J. Hutley L. B.. (2014). Evaluation of the remote-sensing based DIFFUSE model for estimating photosynthesis of vegetation. Remote Sens. Environ. 155, 349–365. doi: 10.1016/j.rse.2014.09.007

[B15] Durand M. Murchie E. H. Lindfors A. V. Urban O. Aphalo P. J. Robson T. M. (2021). Diffuse solar radiation and canopy photosynthesis in a changing environment. Agr. Forest Meteorol. 311, 108684. doi: 10.1016/j.agrformet.2021.108684

[B16] Emmel C. D'Odorico P. Revill A. Hörtnagl L. Ammann C. Buchmann N.. (2020). Canopy photosynthesis of six major arable crops is enhanced under diffuse light due to canopy architecture. Global Change Biol. 26, 5164–5177. doi: 10.1111/gcb.15226 32557891

[B17] Ezhova E. Ylivinkka I. Kuusk J. Komsaare K. Vana M. Krasnova A.. (2018). Direct effect of aerosols on solar radiation and gross primary production in boreal and hemiboreal forests. Atmos. Chem. Phys. 18, 17863–17881. doi: 10.5194/acp-2018-694

[B18] Falge E. Baldocchi D. Olson R. Anthoni P. Aubinet M. Bernhofer C.. (2001). Gap filling strategies for defensible annual sums of net ecosystem exchange. Agr. Forest Meteorol. 107, 43–69. doi: 10.1016/S0168-1923(00)00225-2

[B19] Fang J. Y. Liu G. H. Zhu B. Wang X. K. Liu S. H. (2007). Carbon budgets of three temperate forest ecosystems in dongling mt., Beijing, China. Sci. China Ser. D. 50, 92–101. doi: 10.1007/s11430-007-2031-3

[B20] Farquhar G. D. Roderick M. L. (2003). Pinatubo, diffuse light and the carbon cycle. Science 299, 1997–1998. doi: 10.1126/science.1080681 12663904

[B21] Goodrich J. P. Campbell D. I. Clearwater M. J. Rutledge S. Schipper L. A. (2015). High vapor pressure deficit constrains GPP and the light response of NEE at a southern hemisphere bog. Agr. Forest Meteorol. 203, 54–63. doi: 10.1016/j.agrformet.2015.01.001

[B22] Gu L. H. Baldocchi D. Verma S. B. Black T. A. Vesala T. Falge E. M.. (2002). Advantages of diffuse radiation for terrestrial ecosystem productivity. J. Geophys. Res-Atmos. 107, ACL 2-1–ACL 2-23. doi: 10.1029/2001JD001242

[B23] Gu L. H. Fuentes J. D. Shugart H. H. Staebler R. M. Black T. A. (1999). Responses of net ecosystem exchanges of carbon dioxide to changes in cloudiness: results from two north American deciduous forests. J. Geophys. Res-Atmos. 104, 31421–31434. doi: 10.1029/1999JD901068

[B24] Gui X. Wang L. C. Su X. Yi X. P. Chen X. X. Yao R.. (2021). Environmental factors modulate the diffuse fertilization effect on gross primary productivity across Chinese ecosystems. Sci. Total Environ. 793, 148443. doi: 10.1016/j.scitotenv.2021.148443 34171807

[B25] Guo Z. D. Hu H. F. Li P. Li N. Y. Fang J. Y. (2013). Spatio-temporal changes in biomass carbon sinks in china's forests from 1977 to 2008. Sci. China Life Sci. 56, 661–671. doi: 10.1007/s11427-013-4492-2 23722235

[B26] Han J. Y. Zhang L. M. Li S. G. Wen X. F. Li Q. K. Wang H. M. (2019). Effects of sky conditions on net ecosystem productivity of a subtropical coniferous plantation vary from half-hourly to daily timescales. Sci. Total Environ. 651, 3002–3014. doi: 10.1016/j.scitotenv.2018.10.190 30463150

[B27] He M. Z. Ju W. M. Zhou Y. L. Chen J. M. He H. L. Wang S. Q.. (2013). Development of a two-leaf light use efficiency model for improving the calculation of terrestrial gross primary productivity. Agr. Forest Meteorol. 173, 28–39. doi: 10.1016/j.agrformet.2013.01.003

[B28] Hollinger D. Y. Kelliher F. M. Byers J. N. Hunt J. E. McSeveny T. M. Weir P. L. (1994). Carbon dioxide exchange between an undisturbed old growth temperate forest and the atmosphere. Ecology 75, 134–150. doi: 10.2307/1939390

[B29] Huang K. Wang S. Q. Zhou L. Wang H. M. Zhang J. H. Yan J. H.. (2014). Impacts of diffuse radiation on light use efficiency across terrestrial ecosystems based on eddy covariance observation in China. PloS One 9, e110988. doi: 10.1371/journal.pone.0110988 25393629 PMC4230921

[B30] Jing X. Huang J. Wang G. Higuchi K. Bi J. Sun Y.. (2010). The effects of clouds and aerosols on net ecosystem CO_2_ exchange over semi-arid loess plateau of Northwest China. Atmos. Chem. Phys. 10, 8205–8218. doi: 10.5194/acp-10-8205-2010

[B31] June T. Evans J. R. Farquhar G. D. (2004). A simple new equation for the reversible temperature dependence of photosynthetic electron transport: A study on soybean leaf. Funct. Plant Biol. 31, 275–283. doi: 10.1071/FP03250 32688899

[B32] Kanniah K. D. Beringer J. Hutley L. B. (2011). Environmental controls on the spatial variability of savanna productivity in the northern territory, Australia. Agr. Forest Meteorol. 151, 1429–1439. doi: 10.1016/j.agrformet.2011.06.009

[B33] Kanniah K. D. Beringer J. Hutley L. (2012). Control of atmospheric particles on diffuse radiation and terrestrial plant productivity: A review. Prog. Phys. Geog. 36, 210–238. doi: 10.1016/j.agrformet.2013.06.010

[B34] Kanniah K. D. Beringer J. Hutley L. (2013). Exploring the link between clouds, radiation, and canopy productivity of tropical savannas. Agr. Forest Meteorol. 182–183, 304–313. doi: 10.1016/j.agrformet.2013.06.010

[B35] Kim J. Ryu Y. Dechant B. Lee H. Kim H. S. Kornfeld A.. (2021). Solar-induced chlorophyll fluorescence is non-linearly related to canopy photosynthesis in a temperate evergreen needleleaf forest during the fall transition. Remote Sens. Environ. 258, 112362. doi: 10.1016/j.rse.2021.112362

[B36] Knohl A. Baldocchi D. D. (2008). Effects of diffuse radiation on canopy gas exchange processes in a forest ecosystem. J. Geophys. Res-Atmos. 113, G02023. doi: 10.1029/2007JG000663

[B37] Körner C. (1995). Leaf diffusive conductances in the major vegetation types of the globe ecophysiology of photosynthesis (New York: Springer Press).

[B38] Letts M. G. Lafleur L. P. Roulet N. T. (2005). On the relationship between cloudiness and net ecosystem carbon dioxide exchange in a peatland ecosystem. Ecoscience 12, 53–59. doi: 10.2980/i1195-6860-12-1-53.1

[B39] Li C. Jia X. Ma J. Liu P. Yang R. Bai Y. J.. (2020). Linking diffuse radiation and ecosystem productivity of a desert steppe ecosystem. Peer. J. 8, e9043. doi: 10.7717/peerj.9043 32411524 PMC7207212

[B40] Liu L. B. Gudmundsson L. Hauser M. Qin D. Li S. C. Seneviratne S.I..(2020a).Soil moisture dominates dryness stress on ecosystem production globally. Nat. Commun. 11, 4892. doi: 10.1038/s41467-020-18631-1 32994398 PMC7524720

[B41] Liu P. R. Tong X. J. Zhang J. S. Meng P. Li J. Zhang J. R. (2020b). Estimation of half-hourly diffuse solar radiation over a mixed plantation in north China. Renew. Energ. 149, 1360–1369. doi: 10.1016/j.renene.2019.10.136

[B42] Matsuda R. Ohashi-Kaneko K. Fujiwara K. Goto E. Kurata K. (2004). Photosynthetic characteristics of rice leaves grown under red light with or without supplemental blue light. Plant Cell Physiol. 45, 1870–1874. doi: 10.1093/pcp/pch203 15653806

[B43] McMillen R. T. (1988). An eddy correlation technique with extended applicability to non-simple terrain. Bound-Lay. Meteorol. 43, 231–245. doi: 10.1007/BF00128405

[B44] Melnikova I. Sasai T. (2020). Effects of anthropogenic activity on global terrestrial gross primary production. J. Geophys. Res-Biogeo. 125, e2019JG005403. doi: 10.1029/2019JG005403

[B45] Mercado L. M. Bellouin N. Sitch S. Boucher O. Huntingford C. Wild M.. (2009). Impact of changes in diffuse radiation on the global land carbon sink. Nature 458, 1014–1017. doi: 10.1038/nature07949 19396143

[B46] Michaelis L. Menten M. L. (1913). Die kinetik der invertinwirkun. Biochemische. Zeitschrift 49, 333–369.

[B47] Nichol C. J. Hall F. G. Drolet G. G. Coops N. C. Hilker T. (2010). Estimation of light-use efficiency of terrestrial ecosystems from space: A status report. Bioscience 60, 788–797. doi: 10.1525/bio.2010.60.10.5

[B48] Niyogi D. Chang H. I. Saxena V. K. Holt T. Alapaty K. Booker F.. (2004). Direct observations of the effects of aerosol loading on net ecosystem CO_2_ exchanges over different landscapes. Geophys. Res. Lett. 31, L20506. doi: 10.1029/2004GL020915

[B49] Oliphant A. J. Dragoni D. Deng B. Grimmond C. S. B. Schmid H. P. Scottb S. L. (2011). The role of sky conditions on gross primary production in a mixed deciduous forest. Agr. Forest. Meteorol. 151, 781–791. doi: 10.1016/j.agrformet.2011.01.005

[B50] Park S. B. Knohl A. Lucas-Moffat A. M. Migliavacca M. Gerbig C. Vesala T.. (2018). Strong radiative effect induced by clouds and smoke on forest net ecosystem productivity in central Siberia. Agr. Forest Meteorol. 250–251, 376–387. doi: 10.1016/j.agrformet.2017.09.009

[B51] Pei Y. Y. Dong J. W. Zhang Y. Yuan W. P. Doughty R. Yang J. L.. (2022). Evolution of light use efficiency models: Improvement, uncertainties, and implications. Agr. Forest Meteorol. 317, 108905. doi: 10.1016/j.agrformet.2022.108905

[B52] Reich P. B. (2012). Key canopy traits drive forest productivity. Proc. R. Soc. B 279, 2128–2134. doi: 10.1098/rspb.2011.2270 PMC3321697 22279168

[B53] Rocha A. V. Su H. B. Vogel C. S. Schmid H. P. Curtis P. S. (2004). Photosynthetic and water use efficiency responses to diffuse radiation by an aspen-dominated northern hardwood forest. Forest Sci. 50, 793–801. doi: 10.1093/forestscience/50.6.793

[B54] Roderick M. L. Farquhar G. D. Berry S. L. Noble L. R. (2001). On the direct effect of clouds and atmospheric particles on the productivity and structure of vegetation. Oecologia 129, 21–30. doi: 10.1007/s004420100760 28547064

[B55] Sellers P. J. Randall D. A. Collatzm G. J. Berry J. A. Field C. B. Dazlich D. A.. (1996). A revised land surface parameterization (SiB2) for GCMs. part I: Model formulation. J. Climate 9, 676–705. doi: 10.1175/1520-0442(1996)009<0676:arlspf>2.0.co;2

[B56] Shipley B. (2004). A user’s guide to path analysis, structural equations and causal inference (Cambridge: Cambridge University Press).

[B57] Spitters C. J. T. (1986). Separating the diffuse and direct component of global radiation and its implications for modeling canopy photosynthesis, part i. components of incoming radiation. Agr. Forest Meteorol. 38, 217–229. doi: 10.1016/0168-1923(86)90061-4

[B58] Stanhill G. Cohen S. (2001). Global dimming: A review of the evidence for a widespread and significant reduction in global radiation with discussion of its probable causes and possible agricultural consequences. Agr. Forest Meteorol. 107, 255–278. doi: 10.1016/S0168-1923(00)00241-0

[B59] Steiner A. L. Chameides W. L. (2005). Aerosol-induced thermal effects increase modelled terrestrial photosynthesis and transpiration. Tellus B. 57, 404–411. doi: 10.1111/j.1600-0889.2005.00158.x

[B60] Tong X. J. Meng P. Zhang J. S. Li J. Zheng N. Huang H. (2012). Ecosystem carbon exchange over a warm-temperate mixed plantation in the lithoid hilly area of the north China. Atmos. Environ. 49, 257–267. doi: 10.1016/j.atmosenv.2011.11.049

[B61] Tong X. J. Zhang J. S. Meng P. Li J. Zheng N. (2014). Ecosystem water use efficiency over a warm-temperate mixed plantation in the hilly area of the north China. J. Hydrol. 512, 221–228. doi: 10.1016/j.jhydrol.2014.02.042

[B62] Urban O. Klem K. Ač A. Havránková K. Holišová P. Navrátil M.. (2012). Impact of clear and cloudy sky conditions on the vertical distribution of photosynthetic CO_2_ uptake within a spruce canopy. Funct. Ecol. 26, 46–55. doi: 10.1111/j.1365-2435.2011.01934.x

[B63] Wang K. C. Dickinson R. E. Liang S. L. (2008). Observational evidence on the effects of clouds and aerosols on net ecosystem exchange and evapotranspiration. Geophys. Res. Lett. 35, L10401. doi: 10.1029/2008GL034167

[B64] Wang S. Q. Huang K. Yan H. Yan H. M. Zhou L. Wang H. M.. (2015). Improving the light use efficiency model for simulating terrestrial vegetation gross primary production by the inclusion of diffuse radiation across ecosystems in China. Ecol. Complex. 23, 1–13. doi: 10.1016/j.ecocom.2015.04.004

[B65] Wang S. Ibrom A. Bauer-Gottwein P. Garcia M. (2018). Incorporating diffuse radiation into a light use efficiency and evapotranspiration model: An 11-year study in a high latitude deciduous forest. Agr. Forest. Meteorol. 248, 479–493. doi: 10.1016/j.agrformet.2017.10.023

[B66] Wang S. Zhang L. Huang C. Qiao N. (2017). An NDVI-based vegetation phenology is improved to be more consistent with photosynthesis dynamics through applying a light use efficiency model over Boreal high-latitude forests. Remote Sen. 9, 695. doi: 10.3390/rs9070695

[B67] Webb E. K. Pearman G. I. Leuning R. (1980). Correction of flux measurements for density effects due to heat and water vapour transfer. Q. J. Roy. Meteor. Soc. 106, 85–100. doi: 10.1002/qj.49710644707

[B68] Wu G. H. Jiang C. Y. Kimm H. Wang S. Bernacchi C. Moore C. E.. (2022). Difference in seasonal peak timing of soybean far-red SIF and GPP explained by canopy structure and chlorophyll content. Remote Sens. Environ. 279, 113104. doi: 10.1016/j.rse.2022.113104

[B69] Wu J. van der Linden L. Lasslop G. Carvalhais N. Pilegaard K. Beier C.. (2012). Effects of climate variability and functional changes on the interannual variation of the carbon balance in a temperate deciduous forest. Biogeosciences 9, 13–28. doi: 10.5194/bg-9-715-2012

[B70] Xue W. Lindner S. Nay-Htoon B. Dubbert M. Otieno D. Ko J.. (2016). Nutritional and developmental influences on components of rice crop light use efficiency. Agr. Forest Meteorol. 223, 1–16. doi: 10.1016/j.agrformet.2016.03.018

[B71] Xu H. Zhang Z. Q. Chen J. Q. Xiao J. F. Zhu M. X. Kang M. C.. (2018). Regulations of cloudiness on energy partitioning and water use strategy in a riparian poplar plantation. Agr. Forest Meteorol. 262, 135–146. doi: 10.1016/j.agrformet.2018.07.008

[B72] Xu H. Zhang Z. Chen J. Zhu M. Kang M. (2017). Cloud regulations on the gross primary productivity of a poplar plantation under different environmental conditions. Can. J. Forest Res. 47, 648–658. doi: 10.1139/cjfr-2016-0413

[B73] Yamori W. Hikosaka K. Way D. A. (2014). Temperature response of photosynthesis in C3, C4, and CAM plants: Temperature acclimation and temperature adaptation. Photosynth. Res. 119, 101–117. doi: 10.1007/s11120-013-9874-6 23801171

[B74] Yang X. Y. Asseng S. Wong M. T. F. Yu Q. Li J. Liu E. M. (2013). Quantifying the interactive impacts of global dimming and warming on wheat yield and water use in China. Agr. Forest Meteorol. 182–183, 342–351. doi: 10.1016/j.agrformet.2013.07.006

[B75] Yang X. Y. Li J. Yu Q. Ma Y. C. Tong X. J. Feng Y.. (2019). Impacts of diffuse radiation fraction on light use efficiency and gross primary production of winter wheat in the north China plant. Agr. Forest Meteorol. 275, 233–242. doi: 10.1016/j.agrformet.2019.05.028

[B76] Yang K. G. Ryu Y. Dechant B. Berry J. A. Hwang Y. Jiang C.. (2018). Sun-induced chlorophyll fluorescence is more strongly related to absorbed light than to photosynthesis at half-hourly resolution in a rice paddy. Remote Sens. Environ. 216, 658–673. doi: 10.1016/j.rse.2018.07.008

[B77] Yan H. Wang S. Q. da Rocha H. R. Rap A. Bonal D. Butt N.. (2017). Simulation of the unexpected photosynthetic seasonality in Amazonian evergreen forests by using an improved diffuse fraction-based light use efficiency model. J. Geophys. Res-Biogeo. 112, 3014–3030. doi: 10.1002/2017JG004008

[B78] Yan H. Wang S. Q. Wang J. B. Shugart H. H. (2020). Changes of light components and impacts on interannual variations of photosynthesis in China over 2000–2017 by using a two-leaf light-use-efficiency model. J. Geophys. Res-Biogeo 125, e2020JG005735. doi: 10.1029/2020JG005735

[B79] Yuan W. Cai W. W. Xia J. Z. Chen J. Q. Liu S. G. Dong W. J.. (2014). Global comparison of light use efficiency models for simulating terrestrial vegetation gross primary production based on the LaThuile database. Agr. Forest Meteorol. 192, 108–120. doi: 10.1016/j.agrformet.2014.03.007

[B80] Yuan W. P. Zheng Y. Piao S. L. Ciais P. Lombardozzi D. Wang Y. P.. (2019). Increased atmospheric vapor pressure deficit reduces global vegetation growth. Sci. Adv. 5, eaax1396. doi: 10.1126/sciadv.aax1396 31453338 PMC6693914

[B81] Zhang Z. Y. Liu Q. Z. Ruan Y. C. Tan Y. H. (2021). Estimation of aerosol radiative effects on terrestrial gross primary productivity and water use efficiency using process-based model and satellite data. Atmos. Res. 247, 105245. doi: 10.1016/j.atmosres.2020.105245

[B82] Zhang M. Yu G. R. Zhuang J. Gentry R. Fu Y. L. Sun X. M.. (2011). Effects of cloudiness change on net ecosystem exchange, light use efficiency and water use efficiency in typical ecosystems of China. Agr. Forest Meteorol. 151, 803–816. doi: 10.1016/j.agrformet.2011.01.011

[B83] Zhou H. Yue X. Lei Y. D. Zhang T. Y. Tian C. G. Ma Y. M.. (2021). Responses of gross primary productivity to diffuse radiation at global FLUXNET sites. Atmos. Environ. 244, 117905. doi: 10.1016/j.atmosenv.2020.117905

